# The role of EDTA on rutile flotation using Al^3+^ ions as an activator

**DOI:** 10.1039/c7ra10576b

**Published:** 2018-01-26

**Authors:** Wei Xiao, Chaojun Fang, Jun Wang, Qiannan Liang, Pan Cao, Xingxing Wang, Lijuan Zhang, Guanzhou Qiu, Jun Hu

**Affiliations:** School of Minerals Processing & Bioengineering, Central South University Changsha 410083 China wjwq2000@126.com; Key Lab of Biohydrometallurgy of Ministry of Education Changsha 410083 China; Key Laboratory of Interfacial Physics and Technology, Shanghai Institute of Applied Physics, Chinese Academy of Sciences Shanghai 201800 China zhanglijuan@sinap.ac.cn

## Abstract

Rutile is a relatively stable mineral, due to its weakly specific adsorption to collectors. The development and application of a high efficiency and low-toxicity activator is the main challenge in rutile flotation. In this study, Al^3+^ ions and EDTA (ethylene diamine tetraacetic acid) as mixed activators were investigated using micro-flotation tests. The results of the micro-flotation tests showed that the flotation recovery was slightly increased (from 65.8% to 69.7%) using single Al^3+^ ions as the activator and surprisingly, the activating effect was sharply improved (from 69.7% to 80.6%) after adding EDTA. The activating mechanism of Al^3+^ ions and EDTA was revealed by adsorption capacity measurements, zeta potential measurements, FT-IR spectroscopy analysis and XPS analysis. Al^3+^ ions were adsorbed on the rutile surface in the form of Al(OH)_*n*_^3−*n*^ (*n* = 0, 1, 2), which increased the zeta potential and the activating sites for anionic collector adsorption. The addition of EDTA removed the surplus Al^3+^ ions, and prevented the generation of hydrophilic colloidal Al(OH)_3_ over the pH range of optimum flotation.

## Abstract 2

The adsorption model of Al^3+^ ions, EDTA and SPA on the rutile surface.

## Graphical abstract

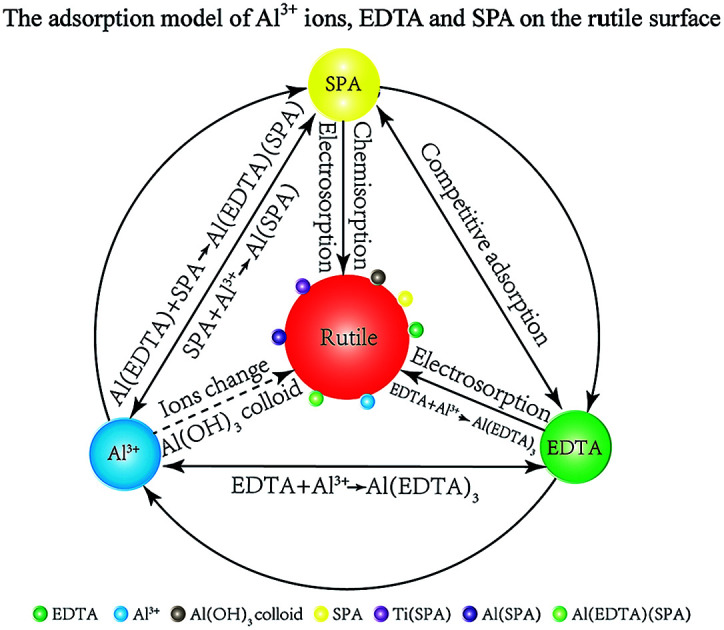

## 1. Introduction

Rutile is the best raw material for high-end titanium pigment production and a high-grade titanium extraction, which has important applications in the defense industry and the high-end coating market.^1,2^ Most rutile ores are refractory ores and its utilization is very difficult due to the fine grain sizes associated with the gangues, the complexity of the mineralogy, and the brittleness that can easily lead to over-grinding.^3^ Although concentration of rutile ores is performed by a combination of gravity, magnetic, flotation and electrostatic separation techniques, flotation is one of the most efficient methods to solve the above issue.

Many researchers have focused on the development and choice of collectors in the process of rutile flotation. A series of collectors, such as fatty acid, benzyl arsenate, hydroxamic acid, organic phosphonate acid, and alkyl dimethyl amine bisphosphonate, are used in laboratory experiments and industrial production.^4–10^ Unfortunately, not one of these collectors can meet the demand of collecting ability, while providing better selectivity. Fatty acid has a strong collecting ability for rutile and gangue minerals, such as amphibole and andradite. Chelating collectors, such as benzyl arsenate, hydroxamic acid, and organic phosphonate acid, exhibit better selectivity, but the collecting ability is very poor due to the lack of activating sites on the rutile surface. A large amount of collector consumption limits the development and utilization of primary rutile.^11^

Rutile is a relatively stable mineral due to the weakly specific adsorption to collectors. The surface modification of the mineral makes it easy for strong adsorption with the chelating collector and increases the hydrophobicity of the mineral surface. Solving the active flotation of minerals containing titanium is a hot topic.^9,11–15^ However, relatively few activating flotations of rutile have been reported. Li *et al.*^15^ used Pb^2+^ ions as an activator for rutile flotation and found that the adsorption of salicyl hydroxamic acid (SHA) on the rutile surface and the flotability of rutile were significantly improved with the addition of Pb^2+^ ions. The activating mechanism explained by them was that this improvement was attributed to the specific adsorption of Pb^2+^ ions in the form of Pb(OH)^+^, which interacted with the Ti–OH group on the rutile surface and formed a surface Ti–O–Pb^+^ complex. Xiao *et al.*^11^ found that Bi^3+^ ions could activate rutile flotation in a strong acidic environment. The mechanism was that Bi^3+^ ions increased the activating sites through the formation of hydroxyl species adsorbing on the rutile surface and then, the proton substitution reaction occurred between the hydroxyl species of Bi^3+^ ions (Bi(OH)_3_^(3−*n*)+^) and the hydroxylated rutile surface (Ti–OH group), producing Ti–O–Bi^2+^ compounds. Although Pb^2+^ and Bi^3+^ ions have a significant activation of rutile flotation, they are still limited in a large number of applications because they belong to the heavy metal ions. In order to reduce pollution of groundwater and rivers from heavy metal ions, new ionic activators must be developed.

In this study, the flotation behavior of rutile using EDTA (ethylene diamine tetraacetic acid) and Al^3+^ ions as the fixed activators and SPA (styryl phosphoric acid) as the collector is investigated using micro-flotation tests. The adsorption and activation mechanisms of Al^3+^ ions and EDTA on the rutile surface were revealed by adsorption capacity measurements, zeta potential measurements, FT-IR and XPS analysis. The presented results are expected to be useful for the development and selection of high efficiency and low-toxicity activators for rutile flotation.

## 2. Materials and methods

### 2.1. Materials and reagents

**Fig. 1:**
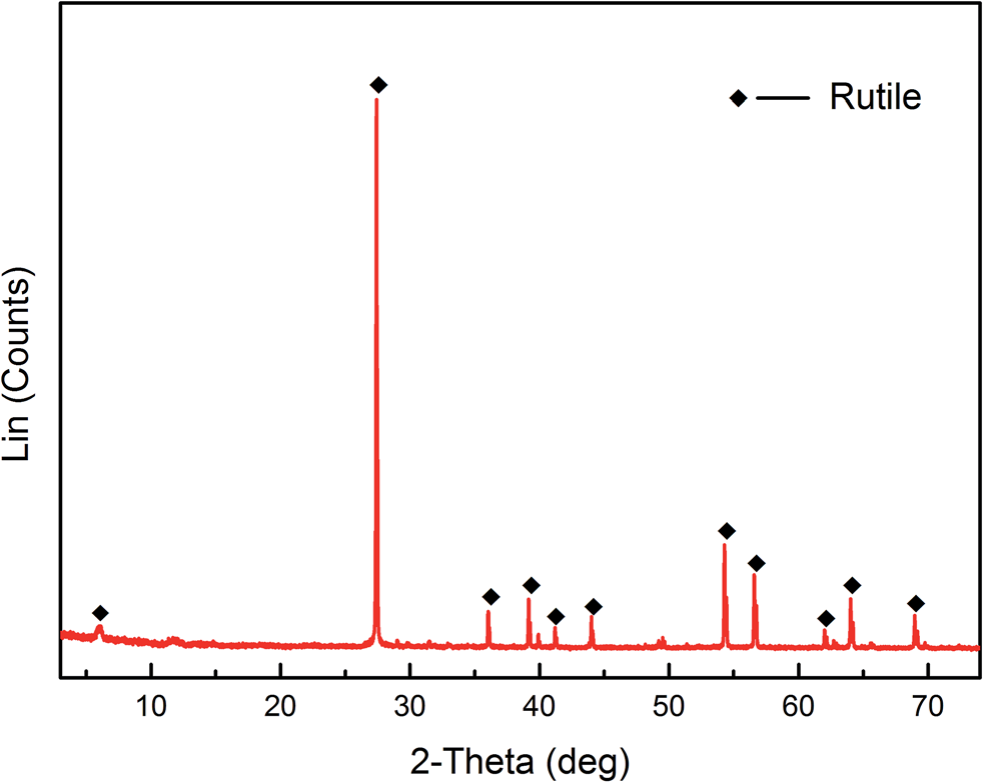
XRD pattern of rutile.

Pure rutile samples were obtained from the Zaoyang rutile mine in Hubei province, China. The samples were ground using an agate mortar. The products were dry sieved to obtain a particle size range of 38 < *d* < 75 μm. A portion of the fraction (*d* < 38 μm) was further ground using an agate mortar and then micro-sieved to obtain a particle size less than 5 μm. The coarse fraction (38 < *d* < 75 μm) was used for micro-flotation tests, adsorption measurements, FT-IR and XPS analysis. The finer fraction (*d* < 5 μm) was used for zeta potential measurements. The results of XRD and XRF are shown in Fig. 1 and Table 1, respectively. The XRD results demonstrated that the pure rutile samples were primarily composed of rutile, and titanium element only originated from the rutile mineral. Multi-element analysis (XRF) of the rutile mineral indicated that the purity of rutile mineral in the sample is calculated to be 93.8%.

**Table 1:** Multi-element analysis of pure rutile mineral (%)

Element	TiO_2_	FeO	Fe_2_O_3_	SiO_2_	CaO	MgO	Al_2_O_3_
Content	93.80	1.53	1.33	2.17	0.17	0.31	0.99

SPA (C_6_H_5_(CH)_2_PO_3_H_2_) was used as the anionic collector for rutile flotation. Aluminum sulfate (Al_2_(SO_4_)_3_·18H_2_O) and EDTA ((HOOCCH_2_)_2_N(CH_2_)_2_N(CH_2_COOH)_2_) were purchased from Tianjin Kemiou Chemical Reagent Co., Ltd. Sodium hydroxide (NaOH) and hydrochloric acid (HCl) were prepared as 0.1% and 10% solutions for pH adjustment of the suspension; the pH of the suspension was monitored using a digital pH meter. The reagents used in the process of measurements and analysis were all analytical grade and deionized water was used in all the experiments.

### 2.2. Micro-flotation experiments

**Fig. 2:**
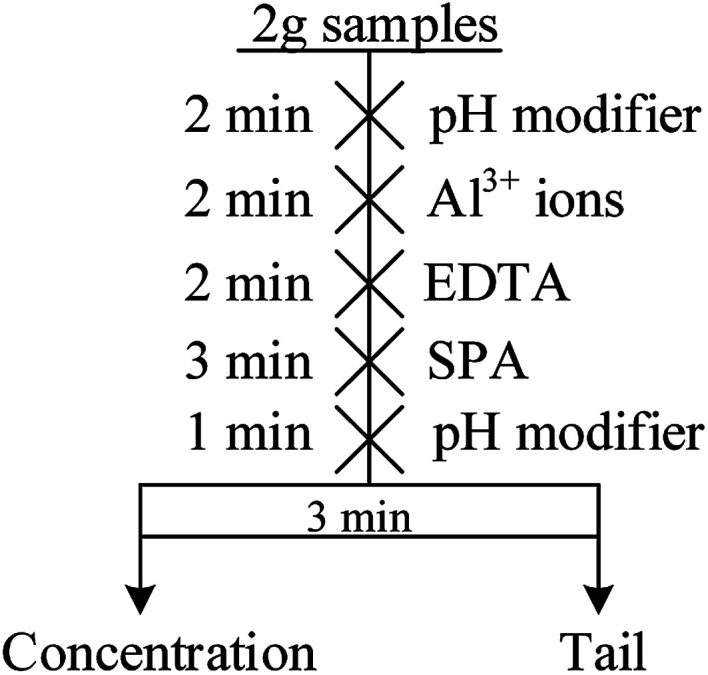
The circuit of pure mineral flotation.

The micro-flotation experiments were carried out in a 40 mL-Plexiglas cell. The pure mineral particles (2.0 g) were placed in a Plexiglas cell, which was filled with 30 mL deionized water. The circuit for the pure mineral flotation is shown in Fig. 2. The pH of the suspension was adjusted by adding HCl or NaOH. After 2 min, Al^3+^ ions, EDTA and SPA were added in sequence. The suspension was agitated for 2, 2 and 3 min, in sequence, between additions. The pH of the suspension was measured and then, the flotation was conducted for 3 min. After filtration, the filtrate was weighed and dried. The mineral recovery was calculated using eqn (1) from the dry weights of the froth concentrates (*m*_1_) and tails (*m*_2_). The results of each micro-flotation test were measured three times, with the average reported as the final value.1
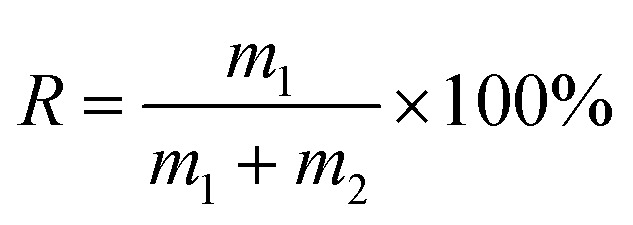
where, *R* is the mineral flotation recovery (%), *m*_1_ is the weight of froth concentration (g), *m*_2_ is the weight of tail (g).

### 2.3. The adsorption capacity measurements

The adsorption capacity was monitored using an Elementar liquid TOCII (German, Elementar Co.). The preparation of slurry solution for adsorption capacity measurement is the same as that of the micro-flotation. After allowing the suspension to settle for 30 min, the liquid portion that separated from the slurry was collected for adsorption measurements. The residual concentration (*C*_e_) of SPA was determined from the standard curve, and the adsorption capacity was calculated by eqn (2) using the residual concentration.2
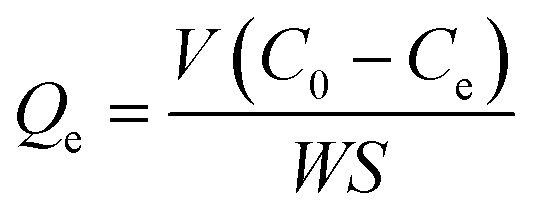
where, *Q*_e_ is the amount of SPA adsorbed on the rutile surface (mol m^−2^), *C*_0_ is the initial concentration of SPA (mol L^−1^), *V* is the volume (L), *S* is the specific surface area (measured by BET) of rutile (m^2^ g^−1^), and *W* is the mass of rutile (g).

### 2.4. Zeta-potential measurements

Zeta potential measurements were carried out on dilute dispersions of pure minerals of particle size less than 5 μm using a Malvern Zeta sizer 4. It measures the electrophoretic shift, which was used to calculate the zeta potential *via* Smoluchowski's equation.^16^ Because its measuring principle is that electrophoresis of mineral particles in solution is performed using a microelectrophoresis apparatus and an electric field is established through the electrodes in the electro compartment, all the measurements were carried out using 0.1 mol L^−1^ KNO_3_ aqueous solution as the background. Suspensions (0.005% mass fraction) of the minerals were dispersed by magnetic stirring for 10 min at room temperature in the presence of various reagents, which were added with the flotation circuit (Fig. 2). After 20 min of settling, the pH of the suspension was measured and the supernatant was obtained for zeta potential measurements. The average value of three replicate measurements for each sample was reported as the final value.

### 2.5. FT-IR spectroscopy

The SHIMADZU IR Affinity-IS Fourier transform infrared spectrometer was used with a KBr disk that contained 0.5% of the required sample and scanned over the wavenumber range of 4000 to 500 cm^−1^ at a resolution of 4 cm^−1^. To prepare the samples for FT-IR analysis, the Al^3+^ ions, EDTA and SPA were 20 times the dosage, which were respectively added in the micro-flotation process, and the time and order for their addition was the same as the micro-flotation. The pH of the slurry solution was controlled at 2.5 ± 0.1. Finally, the solid samples were washed three times using deionized water with the same pH and allowed to dry at room temperature for FT-IR analyses.

### 2.6. XPS experiments

X-ray photoelectron spectroscopy (XPS) measurements were carried out with the model ESCALAB 250Xi. Spectra were recorded at constant pass energy of 20 eV and energy step size of 0.1 eV with Al Kα X-ray as the source. Binding energy calibration was based on C 1s at 284.6 eV. XPS Avantage 5.52 software was used to fit the XPS peaks. The preparation of rutile sample for XPS measurements was similar to that for FT-IR. Subsequently, dry samples were transferred to the spectrometer in an argon atmosphere before the tests.

## 3. Results and discussion

### 3.1. Micro-flotation experiments

**Fig. 3:**
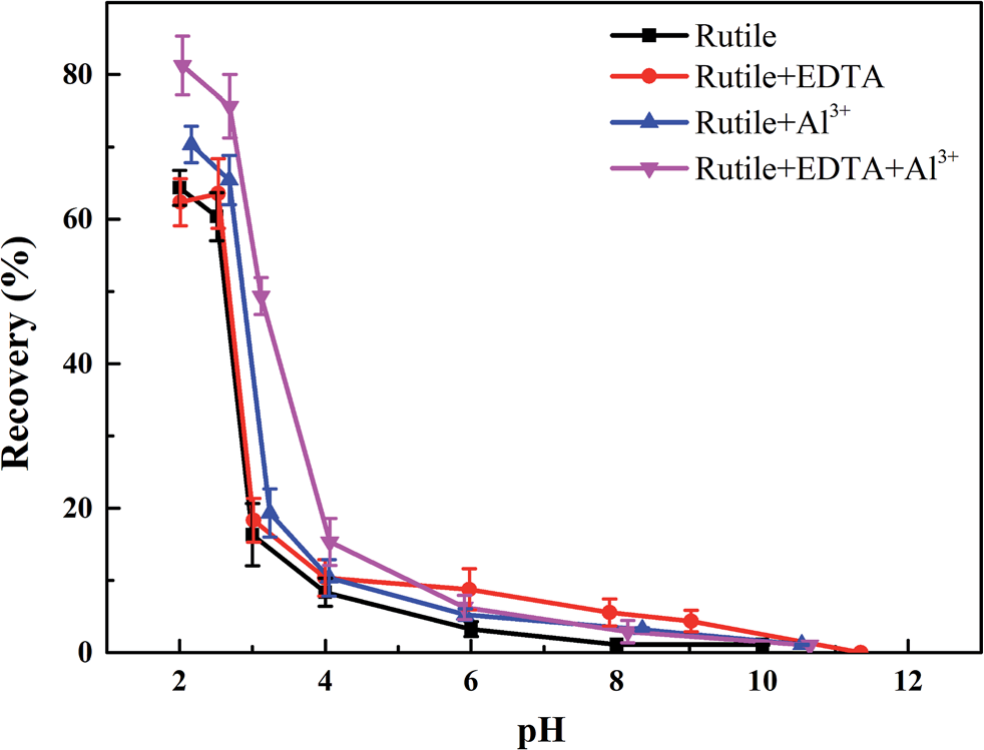
The flotation recoveries of rutile as a function of pH: *c*[SPA] = 5 × 10^−4^ mol L^−1^, *c*[Al^3+^] = 5 × 10^−5^ mol L^−1^ and *c*[EDTA] = 10^−5^ mol L^−1^.

The objective of this study was to investigate the effects of Al^3+^ and EDTA on rutile flotation. The flotation recovery of rutile as a function of pH with 5 × 10^−4^ mol L^−1^ SPA is shown in Fig. 3. As shown in Fig. 3, the four flotation recovery curves rapidly decreased with the increase in the pH value. The best flotation pH range was 2–2.7, which was the natural pH of the SPA solution. The flotation recovery of rutile in the absence Al^3+^ and EDTA reached a maximum value (around 65.8%) at pH 2.2. The flotation recovery of rutile slightly increased after adding only Al^3+^ ions (from 65.8% to 69.7%). In the presence of EDTA and without Al^3+^ ions, the flotation recovery of rutile did not change significantly. This indicated that there was no effect of single EDTA to activate rutile flotation. When Al^3+^ ions and EDTA were added to the slurry solution in sequence, the recovery of rutile sharply increased (from 69.7% to 80.6%). This suggested that there was a synergistic effect between Al^3+^ ion and EDTA; this effect could dramatically improve the adsorption capacity of SPA on the rutile surface. The increase in the adsorption capacity of SPA could directly lead to an increase in the recovery of flotation.^17–19^

### 3.2. The capacity of adsorption measurements

**Fig. 4:**
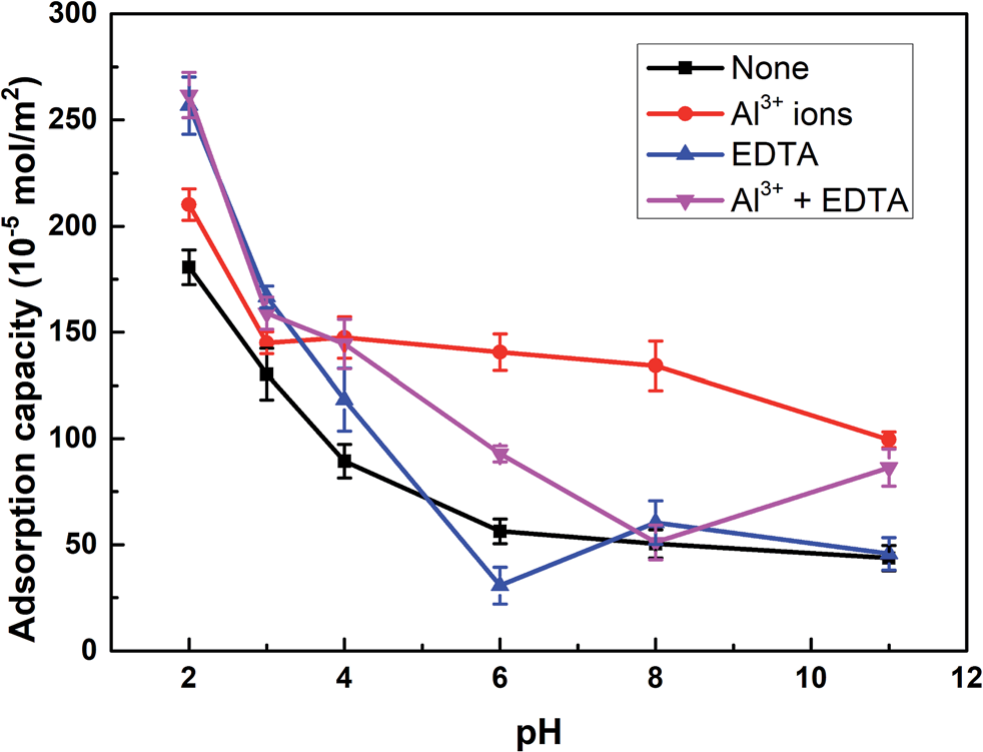
The adsorption capacity of SPA on the rutile surface as a function of pH: *c*[SPA] = 5 × 10^−4^ mol L^−1^, *c*[Al^3+^] = 5 × 10^−5^ mol L^−1^ and *c*[EDTA] = 10^−5^ mol L^−1^.

The adsorption capacity of SPA on rutile surface as a function of pH in the absence and presence of Al^3+^ ions with and without EDTA is shown in Fig. 4. Fig. 4 showed that the adsorption capacity of SPA on the rutile surface decreased with an increase in the pH value. This was caused by the fact that in the slurry solution, there was a competition on the rutile surface between SPA anions and OH^−^ ions.^20^ The higher the pH value, the stronger was the competition. When only Al^3+^ ions were added, the adsorption capacity of SPA slightly increased over the optimal pH range (2–3) of rutile flotation and the adsorption capacity sharply increased when the pH was over 3. When EDTA was added, the adsorption capacity of SPA dramatically increased over the pH range of 2–3, which was the optimal range of pH. This indicated that EDTA could significantly increase the adsorption of SPA on the rutile surface. This was the reason that the recovery of rutile flotation increased in the presence of EDTA.

### 3.3. Zeta-potential measurements

**Fig. 5:**
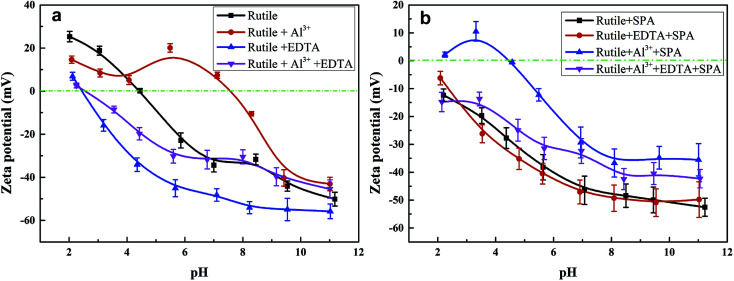
Zeta potential of rutile surface as a function of pH, *c*[SPA] = 5 × 10^−4^ mol L^−1^, *c*[Al^3+^] = 5 × 10^−5^ mol L^−1^ and *c*[EDTA] = 10^−5^ mol L^−1^: (a) without SPA; (b) with SPA.

**Fig. 6:**
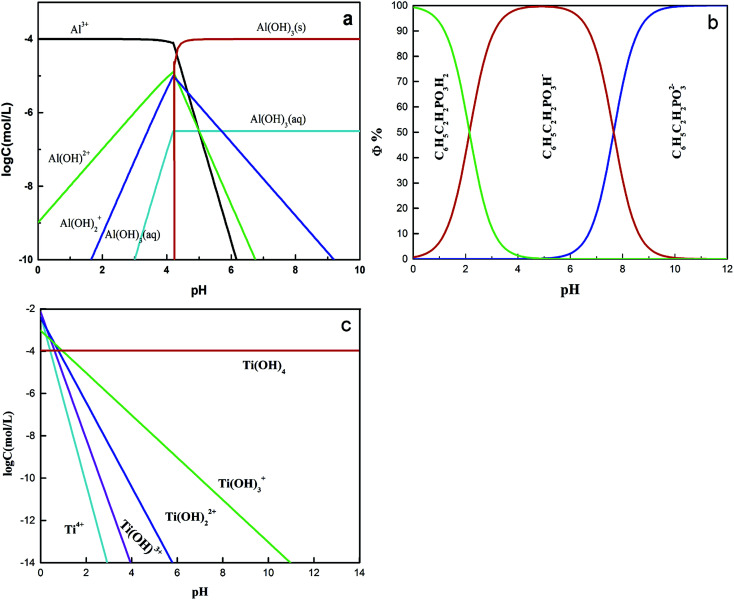
The concentration of each aluminum species (a), each SPA species (b) and each titanium species (c) as a function of pH: *c*[Al] = 10^−4^ mol L^−1^; *c*[SPA] = 5 × 10^−4^ mol L^−1^ and *c*[Ti] = 10^−4^ mol L^−1^.

Zeta potentials of the rutile surface as a function of pH without and with SPA are shown in Fig. 5 and those of aluminum, SPA and titanium species in aqueous solution as a function of pH value are shown in Fig. 6. As shown in Fig. 5a, the isoelectric point (IEP) of rutile particles in the aqueous solution appeared at about 4.4 ± 0.1, which was in accordance with the previous values reported by Parks and Graham.^5,21^ When only Al^3+^ ions were added to the solution, the zeta potential decreased over the pH range of 2–4. The forms of Al^3+^ ions in the aqueous solution were Al^3+^, Al(OH)^2+^, Al(OH)_2_^+^ and a little amount of Al(OH)_3(aq)_ over the pH range of 2–4 (Fig. 6a). If Al^3+^ ions in any form were adsorbed on the rutile surface over this pH range, the zeta potential of the rutile surface would increase. However, it was observed in the measurements that the zeta potential of the rutile surface began to decrease after adding Al^3+^ ions over the pH range of 2–4, which indicated that Al^3+^ ions were not adsorbed on the rutile surface over this pH range. The decrease in the zeta potential was caused by the addition of salt in the aqueous solution. It was reported that the thickness of the electric double layer was compressed by adding salt in the aqueous solution, which led to a decrease in the zeta potential of mineral surfaces.^22–24^ When the pH value was over 4.2, the zeta potential began to rise and the IEP appeared at pH 7.8 ± 0.1. This indicated that Al^3+^ ions were adsorbed on the rutile surface with one or multiple forms of Al^3+^, Al(OH)^2+^, Al(OH)_2_^+^ and Al(OH)_3(aq)_ over this pH range. Al^3+^ ions existed in the aqueous solution in the form of a hydroxyl compound at pH of over 4.2 (in Fig. 6a). This indicated that Al^3+^ ions in the form of hydroxyl compounds were adsorbed on the rutile surface at pH of over 4.2. When only EDTA was added to the solution, the zeta potential of the rutile surface decreased sharply, which was consistent with a previous study.^25^ This indicated that EDTA in an anionic form was adsorbed on the rutile surface. When both Al^3+^ ions and EDTA were added to the solution, the zeta potential was located between those of Al^3+^ and EDTA. The zeta potential in the absence of Al^3+^ without EDTA was slightly different as compared to that in the presence of Al^3+^ with EDTA over the pH range of 7–11.5, but only Al^3+^ ions or EDTA could change the zeta potential. This indicated that over this pH range, Al^3+^ ions and EDTA underwent a complexation reaction and the concentration of Al^3+^ ions and EDTA decreased dramatically.

Fig. 5b shows the zeta potential of rutile surface as a function of pH with SPA. The zeta potential clearly decreased in the presence of SPA (compared with Fig. 5a) and the IEP disappeared over the pH range of the tests. This indicated that the zeta potential shifted to the negative direction after the addition of SPA, which was adsorbed on the rutile surface in the form of SPA^−^ ions. The zeta potential sharply increased in the presence of Al^3+^ ions. However, Al^3+^ ions were not adsorbed on the rutile surface in the absence of SPA over the pH range of 2–4. The addition of Al^3+^ ions could increase the zeta potential of the rutile surface in the presence of SPA. This indicated that Al^3+^ chemically reacted with SPA^−^ ions, and produced a phosphonate salt precipitate, which was adsorbed on the rutile surface. EDTA could not clearly decrease the zeta potential of rutile surface in the presence of SPA (Fig. 5b). However, the zeta potential sharply decreased without SPA (Fig. 5a). This indicated that competitive adsorption existed on the rutile surface between SPA and EDTA and consequently, the adsorption ability of SPA became stronger than that of EDTA. When both Al^3+^ ions and EDTA were added to solution in the presence of SPA, the tendency of the zeta potential of the rutile surface was similar to that without Al^3+^ ions over the pH range of 2–4. In addition, as shown in Fig. 6a, when the concentration of Al^3+^ ions was 10^−4^ mol L^−1^, colloidal Al(OH)_3_ began to be generated at pH 3.0. However, when the concentration of Al^3+^ ions was 10^−3^ mol L^−1^, colloidal Al(OH)_3_ began to be generated at pH 2.3. Therefore, the production of colloidal Al(OH)_3_ was avoided in the flotation process by using EDTA to control the concentration of Al^3+^ ions in solution. This suggested that the addition of EDTA removed the surplus Al^3+^ ions in solution and prevented the generation of hydrophilic colloidal Al(OH)_3_.

### 3.4. FT-IR spectroscopy

**Fig. 7:**
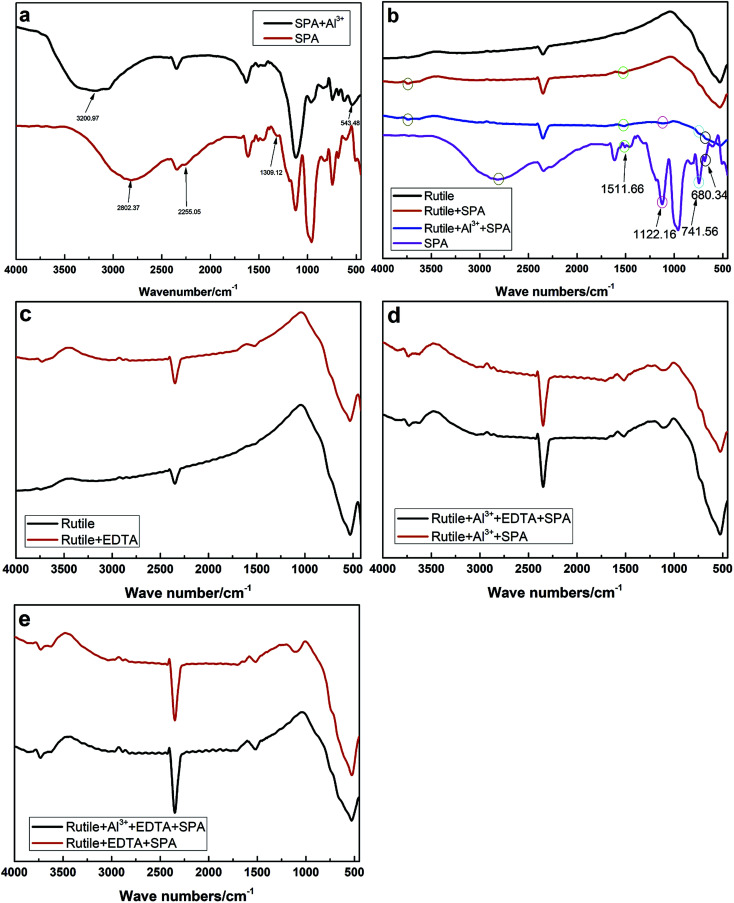
Infrared spectra of SPA and rutile: (a), SPA before and after treated with Al^3+^ ions; (b), SPA and rutile before and after treated with SPA and Al^3+^ ions; (c), rutile before and after treated with EDTA; (d), rutile treated with Al^3+^ ions and SPA with and without EDTA; (e), rutile treated with EDTA and SPA with and without Al^3+^ ions.

The infrared spectra of SPA and rutile at different conditions are shown in Fig. 7. The infrared spectrum of SPA before and after treatment with Al^3+^ ions is shown in Fig. 7a. After the addition of Al^3+^ ions, an additional peak at 543.28 cm^−1^ appeared in the spectrum of SPA. The additional peak can probably be attributed to the Al–O skeleton-vibration, whose peak was reported as appearing at 540–575 cm^−1^.^26^ In addition, the O–H stretching vibration in the spectra shifted from 2802.37 to 3200.97 cm^−1^, indicating that one O–H in an SPA molecule may chemically react with an Al^3+^ ion and consequently stabilize other O–H. Finally, the peak at 610.26 cm^−1^ was associated with the stretching vibrations of the C–P–O group and shifted to 616.24 cm^−1^ after adsorption with Al^3+^ ions. These results indicated that Al^3+^ ions chemically reacted with SPA.

Fig. 7b shows the spectra of the rutile sample in the absence and presence of SPA with and without Al^3+^ ions and the collector single SPA, respectively. The spectra illustrated that the peaks at 610.26, 680.34, 1122.16 and 1511.66 cm^−1^ were characteristic for molecules containing the C–P–O group, C=C bond, P=O bond and benzene ring, respectively. After treatment with SPA, an additional peak at 1511.66 cm^−1^ appeared in the spectrum of rutile, which was the characteristic for molecules containing the benzene ring. This indicated that SPA was adsorbed on the rutile surface *via* chemical adsorption. With the addition of Al^3+^ ions, three new peaks in the spectrum of rutile appeared at 680.34, 741.56 and 1122.16 cm^−1^, which were attributed to SPA, suggesting that Al^3+^ ions could clearly improve the adsorption of SPA on the rutile surface.

The spectrum of rutile after and before treatment with EDTA in the absence and presence of Al^3+^ ions and EDTA are shown in Fig. 7c and d, respectively. In Fig. 7c, the peak appeared at 1492.17 cm^−1^ in the rutile spectrum after EDTA adsorption and was attributed to the scissor bending vibration of the –CH_2_– group in the EDTA molecule. However, it was found that there was no difference after and before treatment with EDTA in the presence of SPA (Fig. 7d). This revealed that EDTA could be adsorbed on the rutile surface, but a strong competitive adsorption existed on the rutile surface between SPA and EDTA. This is very consistent with our previous conclusion from zeta potential measurements.

The spectrum of rutile after and before treatment with Al^3+^ ions in the presence of EDTA and SPA is shown in Fig. 7e. It was found that there were two new peaks located at 1122.13 and 1634.07 cm^−1^ on the spectrum of rutile after treatment with Al^3+^ ions, which were assigned to the stretching vibrations of the P=O bond and the absorption peak of disubstituted –CH=CH–, respectively. In particular, the peak attributed to the stretching vibrations of the P=O bond was moderately strong. This indicated that Al^3+^ could clearly improve the adsorption of SPA on the rutile surface in the presence of EDTA.

### 3.5. XPS experiments

**Fig. 8:**
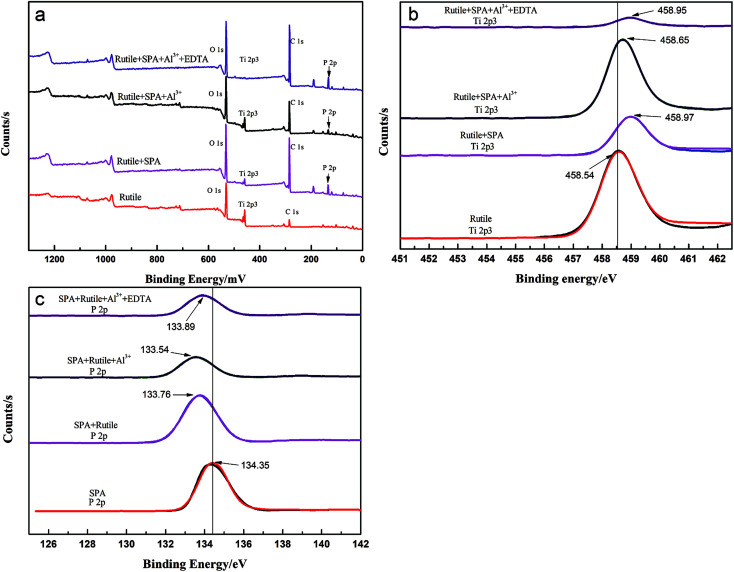
The survey scan and high-resolution xps spectra of rutile.

The chemical composition of the rutile surface before and after Al^3+^ ion treatment was determined using XPS. The survey scan XPS spectra of rutile, rutile with adsorbed SPA, rutile treated with Al^3+^ ions and SPA and rutile treated with Al^3+^ ions, EDTA and SPA over a binding energy range of 1300–0 eV is shown in Fig. 8. The XPS peak of P 2p appeared in the spectrum of rutile treated with SPA, which indicated that SPA chemically reacted with Ti element on the rutile surface. This was strongly consistent with the results of the infrared spectra of rutile adsorbed with SPA. Their atomic concentrations are presented in Table 2. The atomic concentration of P after the addition of Al^3+^ ions decreased, which showed that the adsorption capacity of SPA on the rutile surface decreased in the presence of Al^3+^ ions. The adsorption capacity measurement and the zeta potential measurement also satisfactorily agreed with the XPS results. With the addition of EDTA, the atomic concentration of P sharply increased and reached the highest value. This indicated that Al^3+^ ions were detrimental for the adsorption of SPA, but EDTA could remove the negative effect. The previously performed micro-flotation process showed that Al^3+^ ions could improve the flotation recovery, and the addition of EDTA could increase the flotation recovery sharply. However, the results of the adsorption capacity measurement and XPS analysis showed that the adsorption capacity of SPA on the rutile surface decreased with the addition of Al^3+^ ions. The contradiction between the flotation and the adsorption capacity suggested that a new adsorption state could occur upon the addition of Al^3+^ ions. The new adsorption state should be the coordination adsorption, which is more stable than the common physical adsorption and electrostatic adsorption. Because only the collector formed a more stable adsorption on the mineral surface, the increase in mineral flotation recovery occurred due to the decrease in collector adsorption. Both P and Al atomic concentrations increased with the addition of EDTA, which suggested that EDTA could improve the new adsorption state. This was strongly consistent with the results of the micro-flotation and adsorption capacity measurements.

**Table 2:** Atomic concentration of elements on rutile surface as determined by XPS

Samples^a^	C (%)	O (%)	Ti (%)	P (%)	Al (%)	N (%)
1	25.01	62.50	12.49	—	—	—
2	62.02	28.61	1.57	7.80	—	—
3	51.61	36.06	4.67	4.90	2.77	—
4	62.40	25.16	0.41	8.27	3.64	0.11

a (1)-rutile; (2)-rutile + SPA; (3)-rutile + Al^3+^ ions + SPA; (4)-rutile + Al^3+^ ions + EDTA + SPA.

The peak of Al 2p was traced on the spectra of rutile treated with Al^3+^ ions and SPA. The high-resolution XPS spectra of Ti, P and Al are shown in Fig. 8b and c, respectively. Fig. 8b showed that the Ti 2p_3_ binding energy peaks were positioned at about 458.54, 458.95, 458.65 and 458.97 eV, which was characteristic of rutile titanium dioxide.^27^ After adsorbing SPA, the binding energy peak of Ti 2p_3_ shifted to the higher binging energy, which indicated that the adsorption of SPA could lead to an increase in Ti electronegativity.

Fig. 8c shows that the P 2p XPS bands appeared on the SPA and rutile surfaces. The P 2p XPS bands for SPA were composed of a component at 143.35 eV, which was consistent with metaphosphate. The samples of adsorbed rutile, adsorbed rutile treated with Al^3+^ ions and that subsequently treated with EDTA were analyzed by XPS; shifts in the P 2p peaks towards lower binding energy by about 0.59, 0.81 and 0.46 eV, respectively, were detected. The shifts of the P 2p binding energy suggested a change in the chemical states of the samples.^9^ Thus, the results of the XPS analysis was a clear evidence that the low electronegativity ions (such as Al^3+^ and Ti^4+^ ions) chemically reacted with SPA and produced a metal–SPA complex on the rutile surface, which was consistent with the results of the zeta potential measurements.

**Fig. 9:**
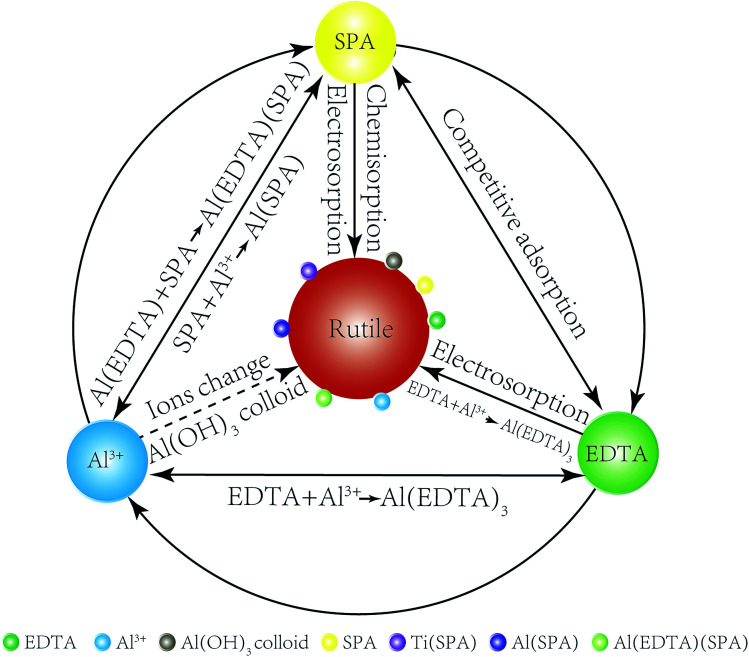
An adsorption model of Al^3+^ ions, EDTA and SPA on the rutile surface.

The adsorption model of Al^3+^ ions, EDTA and SPA on the rutile surface is obtained by the results of adsorption capacity measurements, zeta potential measurements, FT-IR analysis and XPS analysis as follows. In Fig. 9, a comparison of Al^3+^ ions, EDTA, SPA and rutile surface is clearly drawn. SPA could adsorb on the rutile surface through electrosorption and chemisorption, EDTA through electrosorption and Al^3+^ ions by changing ions and formation of Al(OH)_3_ colloid. EDTA could also chemically react with Al(OH)_3_ colloid, which adsorbed on the rutile surface. EDTA and SPA could not chemically react directly, but they could bond together on adding Al^3+^ ions.

## 4. Conclusion

Using Al^3+^ ions and EDTA as a combined activator to float rutile with SPA as the collector was investigated by micro-flotation tests, adsorption capacity measurements, zeta potential measurements, FT-IR spectroscopy and XPS analysis. Based on the experimental results, the following conclusions could be drawn:

(1) The flotation recovery was slightly increased (from 65.8% to 69.7%) using single Al^3+^ ions as the activator and the activating effect was sharply improved (from 69.7% to 80.6%) after adding EDTA at pH of about 2.2.

(2) Al^3+^ ions were adsorbed on the rutile surface in the form of Al(OH)_*n*_^3−*n*^ (*n* = 0, 1, 2), which increased the zeta potential and the active sites for collector adsorption.

(3) The addition of EDTA removed the surplus Al^3+^ ions, and prevented hydrophilic colloidal Al(OH)_3_ generation.

## Conflicts of interest

There are no conflicts of interest to declare.

## Supplementary Material
